# Correlation Analysis of Genetic Mutations and Galectin Levels in Breast Cancer Patients

**DOI:** 10.3390/genes15060818

**Published:** 2024-06-20

**Authors:** Ella G. Markalunas, David H. Arnold, Avery T. Funkhouser, Julie C. Martin, Michael Shtutman, W. Jeffery Edenfield, Anna V. Blenda

**Affiliations:** 1 Department of Public Health, Brown University, Providence, RI 02912, USA; ella_markalunas@brown.edu; 2 Department of Biomedical Sciences, University of South Carolina School of Medicine Greenville, Greenville, SC 29605, USA; dharnold@email.sc.edu (D.H.A.); averytf@email.sc.edu (A.T.F.); 3 Prisma Health Cancer Institute, Prisma Health, Greenville, SC 29605, USA; julie.martin@prismahealth.org (J.C.M.); jeffery.edenfield@prismahealth.org (W.J.E.); 4 Department of Drug Discovery and Biomedical Sciences, University of South Carolina College of Pharmacy, Columbia, SC 29208, USA; shtutmanm@cop.sc.edu; 5 Department of Medicine, University of South Carolina School of Medicine Greenville, Greenville, SC 29605, USA

**Keywords:** breast cancer, genetic mutation, biomarker, galectins, metastasis

## Abstract

Galectins are innate immune system regulators associated with disease progression in cancer. This paper aims to investigate the correlation between mutated cancer-critical genes and galectin levels in breast cancer patients to determine whether galectins and genetic profiles can be used as biomarkers for disease and potential therapy targets. Prisma Health Cancer Institute’s Biorepository provided seventy-one breast cancer samples, including all four stages spanning the major molecular subtypes and histologies. Hotspot mutation statuses of cancer-critical genes were determined using multiplex PCR in tumor samples from the same patients by Precision Genetics and the University of South Carolina Functional Genomics Core Facility. The galectin-1, -3, and -9 levels in patients’ sera were analyzed using Enzyme-linked Immunosorbent Assay (ELISA). An analysis was performed using JMP software to compare mean and median serum galectin levels between samples with and without specific cancer-critical genes, including pooled *t*-test, Wilcoxon Rank Sum Test, ANOVA, and Steel Dwass Test (α=0.05). Our analysis indicates that *KIT* mutations correlate with elevated serum levels of galectin-9 in patients with breast cancer. In patients with Luminal A subtype, *FLT3* mutation correlates with lower serum galectin-1 and -9 levels and *TP53* mutations correlate with higher serum galectin-3 levels. Patients with invasive ductal carcinoma had significantly higher serum galectin-3 levels than patients with ductal carcinoma in situ. Patients with both *TP53* and *PIK3CA* mutations exhibit elevated serum galectin-3 levels, while patients with one or neither mutation show no significant difference in serum galectin-3 levels. In addition, metastatic breast cancer samples were more likely to have a *KIT* or *PIK3CA* mutation compared to primary breast cancer samples. The relationship between genetic mutations and galectin levels has the potential to identify appropriate candidates for combined therapy, targeting genetic mutations and galectins. Further understanding of the effect of genetic mutations and galectin levels on cancer progression and metastasis could aid in the search for biomarkers for breast cancer diagnosis, disease progression, and prognosis.

## 1. Introduction

Breast cancer is one of the most prevalent cancers worldwide. In 2020, it was estimated that 2.3 million women were diagnosed with breast cancer and that is caused 685,000 deaths worldwide [1]. While certain factors including biological sex, age, and family history of breast cancer increase the risk of breast cancer, there are also numerous genetic mutations associated with breast cancer. Mutations in *BRCA1* and *BRCA2* are observed in approximately 25% of breast cancer cases [2]. Other high-penetrance genes that predispose one to breast cancer include *PTEN*, *TP53*, *CDH1*, and *STK11*, though mutations in these genes are rarer compared to *BRCA1* and *BRCA2*. There are also multiple DNA repair genes that interact with the *BRCA* genes such as *ATM*, *CHEK2*, and *BRIP1* that further increase the risk of breast cancer when mutated. For instance, *CHEK2* is a protein kinase and G2 cell cycle regulator that stabilizes p53 when DNA damage occurs and interacts with *BRCA1*. The 1100delC mutation in *CHEK2* has been found to increase the risk of breast cancer two-fold in women and ten-fold in men. Many of the mutated genes analyzed in this study, including *KIT*, *MET*, and *FLT3*, play a role in the Receptor Tyrosine Kinase (RTK) and RAS pathway. Most RTKs possess a heavily glycosylated extracellular N-terminal binding site and an intracellular tyrosine kinase domain [3]. These kinases can activate upon interactions with multiple types of ligands. Often in cancer, mutations of genes encoding RTKs result in a gain of function, thereby facilitating cell proliferation, differentiation, and migration [4].

The most common treatment options for breast cancer include surgery, radiation, and chemotherapy drugs such as anthracyclines, taxanes, and alkylating agents [5]. Current strategies for personalized treatments are based on the molecular subtype of breast cancer, Luminal A, Luminal B, HER2 Enriched, and Triple-Negative (TNBC), which are classified based on the hormone receptors expressed. For instance, trastuzumab is a monoclonal antibody therapy that targets HER2 receptors; however, this drug would not be effective against TNBC. Therapies against TNBC must target other tumor markers such as VEGF with bevacizumab or EGFR with cetuximab. Since many of these drugs are broad-acting, damaging healthy cells in the process and causing various adverse effects, it is desired to develop therapies that are capable of targeting cancer cells. One such target that has gained attention in research is galectins.

Galectins are a family of soluble proteins that are expressed across various cell types and participate in numerous cellular processes, including the regulation of cell growth, apoptosis, cell migration, and immune evasion for tumors [6]. For instance, galectin-1 (gal-1) has been shown to increase the frequency of Foxp3+ T*_reg_* cells in the microenvironment of breast cancer cells in mice, contributing to tumor evasion of the immune system [7]. Similarly, another study found that gal-1 interacts strongly with the N-glycosylated neuropilin-1 domains on PDGFR and TGF-βR. This induced TGF-β and PDGF signaling, promoting the migration and activation of hepatic stellate cells [8]. Looking at other galectins, galectin-3 (gal-3) has demonstrated the ability to prevent nitric oxide-induced apoptosis in human breast carcinoma cells [9]. Zhang et al. found that treating galectin-3 knockdown breast cancer cells with the apoptotic inducer arsenic trioxide increased its apoptotic effects compared to galectin-3-positive breast cancer cells, demonstrating an association between galectin-3 expression and chemotherapeutic resistance [10]. Additionally, galectin-3 can disrupt N-cadherin cell–cell junctions, providing a mechanism to promote tumor cell motility and metastasis [11]. Galectin-9 (gal-9) is peculiar in that studies demonstrate its ability to act as a tumor-promoting and anti-tumor protein. Morishita et al. demonstrated the ability of galectin-9 to promote apoptosis in colon cancer, which they suspected is through the increased phosphorylation of the RTKs ALK, DDR1, and EPHA10 [12]. Meanwhile, galectin-9 has also been shown to bind to Tim-3, a cell surface molecule on Th1 cells that suppresses their immune functions and induces apoptosis [13]. Considering the variety of galectins and their numerous processes in promoting cancer development, the concept of galectin inhibitors sparks interest as a potential therapy.

The development of galectin inhibitors began decades ago when the role of galectins in cancer progression and tumor development was discovered. There are currently two main types of galectin inhibitors in trials: carbohydrate-based and non-carbohydrate-based [14]. Thiodigalactoside, a carbohydrate-based galectin-1 inhibitor, has been shown to prevent angiogenesis and tumor growth while preventing metastasis and inducing the apoptosis of tumor cells in breast cancer samples in tumor mouse models [15]. Anginex is a peptide-based galectin-1 inhibitor that has demonstrated the potential to inhibit tumor growth, proliferation, and angiogenesis [16]. Modified citrus pectin is a carbohydrate-based galectin-3 inhibitor that has also shown antimetastatic properties as well as promise in inhibiting tumor growth and restoring T-cell surveillance [17]. Many studies and early-phase clinical trials are investigating the efficacy of galectin inhibitors with and without other chemotherapies or monoclonal antibodies. These include gene-specific targeted therapies, which have been in practice since the development of imatinib to target *BCR-ABL* in 2001. FDA-approved therapies specific to breast cancer include Olaparib, which has demonstrated clinical efficacy for patients with *ATM*, *BRCA1/2*, and *CHEK2* mutations, as well as alpelisib for patients with *PIK3CA* mutations [18]. In a Phase I clinical trial, GM-CT-01, a galectin-1 and -3 inhibitor, is being tested with and without 5-fluorouracil in patients with advanced-stage solid tumor cancers, including breast cancer to study the effects of galectin inhibitors on disease progression and their ability to improve the chemotherapy response in patients who have not responded well to previous treatments [19]. Another study found that combining a galectin-9 inhibitor with AZD1930, an *ATM* inhibitor, led to decreased tumor growth and significantly longer survival for mouse models [20]. Overall, the incorporation of galectin inhibitors into chemotherapy is promising and their combination with other chemotherapeutic drugs provides an opportunity for clinicians to create more personalized treatment regimens based on the inherent risks and prognosis associated with specific mutations.

With the increasing interest in galectins as a target in cancer therapy, this paper seeks to explore the relationship between cancer-driving mutations in breast cancer patients and serum galectin levels. Additionally, tumor characteristics, including stage and metastasis, were analyzed in relation to galectin levels and mutations. The results of our research could provide new insights into the correlations between specific breast cancer mutations and certain galectins. As galectin inhibitors are employed in conjunction with existing therapies, understanding the relationships between certain mutations and galectins could help produce more personalized therapeutic regimens that improve responsiveness and patient outcomes. Additionally, the correlation between specific genetic mutations and galectin levels will provide crucial insights into ideal candidates for galectin-modulated therapies.

## 2. Materials and Methods

### 2.1. Sample Acquisition

Seventy-one breast cancer patient serum samples were obtained from Prisma Health Cancer Institute’s (PHCI) Biorepository based on specimen and gene panel availability. Patients signed a consent form when the tissue was procured. Patient sample data are included in the Supplementary Materials section.

Thirty-six of the samples were obtained from patients with the Luminal A subtype (50.7%), nine were from patients with the Luminal B subtype (12.7%), six were from patients with the Luminal A HER2 Hybrid subtype (8.5%), two were from patients with the Luminal B HER2 Hybrid subtype (2.8%), two were from the HER2 Positive subtype (2.8%), twelve were from patients with the Triple-Negative subtype (16.9%), and the molecular subtypes of four patients were unknown (5.6%).

Twenty-four of the samples were stage I (33.8%), twenty-eight of the samples were stage II (39.4%), thirteen of the samples were stage III (18.3%), and six of the samples were stage IV (8.5%). Fifty-nine of the samples were from patients with primary breast cancer (83.1%), eight of the samples were from patients with metastatic breast cancer (11.3%), and four of the samples were from patients with recurrent breast cancer (5.6%).

### 2.2. ELISA for Galectin Profiling

The galectin levels of patients’ sera were analyzed using enzyme-linked immunosorbent assay (ELISA). ELISA kits from R&D systems (Minneapolis, MN, USA) were used to measure the concentrations of galectin-1, -3, and -9. The serum concentrations of galectin-1 and -3 were effectively obtained in all seventy-one samples, and the serum concentration of galectin-9 was obtained in fifty samples.

### 2.3. Hotspot Panel for Cancer-Critical Genetic Mutations

Genetic Mutation Data for sixty-five samples was obtained by Precision Genetics as described in “KIT Mutations Correlate with Higher Galectin Levels and Metastasis in Breast and Non-Small Cell Lung Cancer” [21].

Data for an additional six samples were obtained by the University of South Carolina College of Pharmacy Functional Genomics Core Facility. Genomics DNA was purified from frozen tumor samples with QIAGEN DNA Blood and Tissue Kit (#69506, QIAGEN, Carlsbad, CA, USA), and Genomics DNA from FFPE tumor samples was purified with Quick-DNA FFPE MiniPrep Kit (D3067, Zymo Research, Irvine, CA, USA). Cancer hot-spot mutation regions were amplified with CleanPlex OncoZoom Cancer Hot-spot Panel (916001 Paragon Genomics, Hayward, CA, USA). The panel includes 601 amplicons, covering 65 genes.

The amplicons were sequenced with Illumina Novoseq 6000 in a partial lane of the S4 flow cell, PE150, with a sequencing depth of 2 million reads per sample. The sequences were aligned to Paragon Genomics amplicons reference sequences, and the variants were called the GATK pipeline (PMID: 25431634). The biologically and clinically relevant tumor-specific alternation was determined with the Cancer Genome Interpreter (PMID: 29592813) and NCBI ClinVar database (PMID: 29165669).

### 2.4. Data Analysis

JMP was used to perform statistical analyses. JMP Pro 16 is a software by the SAS Institute (Cary, NC, USA). Prism was used to generate the figures included in this paper. Prism is a part of GraphPad Software version 10.2.3 (Boston, MA, USA). The distributions of serum galectin levels of samples with specific genetic mutations were compared using a *t*-test for the difference of means. A Levene’s Test for Homogeneity of Variance was used to determine whether a pooled two-sample *t*-test, which assumes equal variances between the populations, or Welch’s test, which assumes unequal variances between populations, is more appropriate. For tests with small sample sizes (*n* < 10), a Wilcoxon Rank Sum Test was performed to compare medians because the normality of residuals could not be assumed. For tests comparing more than two samples, ANOVA and the Steel Dwass test were used for parametric and nonparametric comparisons, respectively. A *p*-value less than 0.05 was considered to be statistically significant.

## 3. Results

### 3.1. Data Statistic Summary

Table 1 demonstrates the baseline distribution of galectin-1, -3, and -9 for breast cancer patients in our sample.

### 3.2. Mutation Distribution

Table 2 shows the frequencies of specific point mutations. Some samples have multiple hotspot mutations on the same cancer-critical gene. Because of this, the total mutation count is higher than the sample count. Pooled *t*-test results found no statistically significant difference between specific point mutations and galectin levels. Because of this, only whether the gene was mutated or not in the patient sample was used for further statistical analyses.

**Figure 1:**
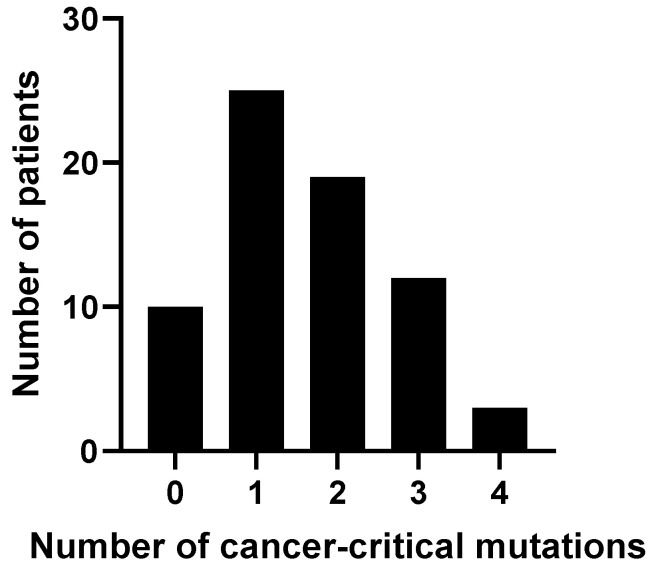
Distribution of frequency of mutations among patient samples.

Figure 1 shows the distribution of the number of cancer-critical gene mutations among patient samples. No significant difference was found in serum galectin levels between cohorts with different numbers of mutated cancer-critical genes.

### 3.3. Correlation between Galectins and Genetic Mutations

A pooled *t*-test was used to analyze the correlation between the presence of a mutation in *PIK3CA*, *TP53*, and *KDR* genes and serum galectin levels. As shown in Table 3, no significant correlation was found between the presence of a *PIK3CA*, *TP53*, and *KDR* mutation and a difference in mean serum galectin levels. The use of a Welch’s *t*-test in place of a pooled *t*-test as indicated by a Levene’s Test is marked by an asterisk (*).

**Figure 2:**
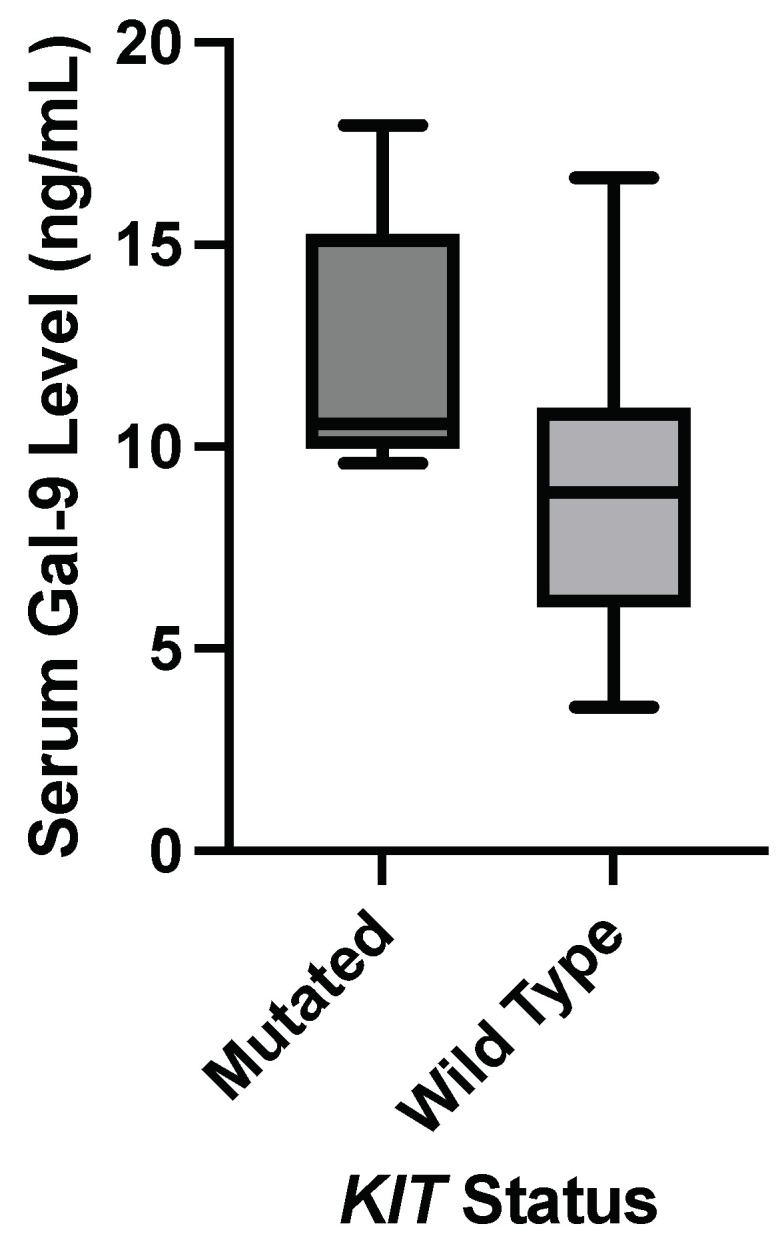
Nonparametric comparison of serum galectin levels of breast cancer patients. Serum galectin-9 levels were significantly elevated in patients with a *KIT* mutation (*p*-value = 0.0485).

As shown in Table 4 and Figure 2, the Wilcoxon Rank Sum Test supports a significant correlation between a mutated *KIT* gene and an elevation of serum galectin-9 levels. No significant correlation was found between a *MET* mutation and a difference in galectin levels.

### 3.4. Molecular Subtypes

Breast cancer molecular subtypes are based on the expression of hormone receptors such as estrogen and progesterone receptors; the presence of additional copies of the gene for Human Epidermal Growth Factor Receptor 2 (HER2); and Ki-67 levels, which serve as a measurement of cellular proliferation [22]. The genes expressed by cancer cells determine the cancer’s molecular subtype and how the cells behave during the course of the disease. Luminal A breast cancer is the most common subtype and is characterized as hormone receptor-positive and HER2-negative, with low levels of Ki-67.

#### 3.4.1. Luminal A

The serum galectin levels of the Luminal A subtype samples (*N* = 36) were analyzed against the presence of specific cancer-critical gene mutations. Table 5 shows the *p*-values for pooled *t*-test comparisons of mean serum galectin levels and the presence of a hotspot mutation in *PIK3CA* and *KDR* genes, which both had mutated and wild-type cohorts that were larger than or equal to ten. The number of mutated samples is out of thirty-six samples classified as Luminal A. No significant correlation was found between a mutation in *PIK3CA* or *KDR* genes and abnormal galectin levels.

**Figure 3:**
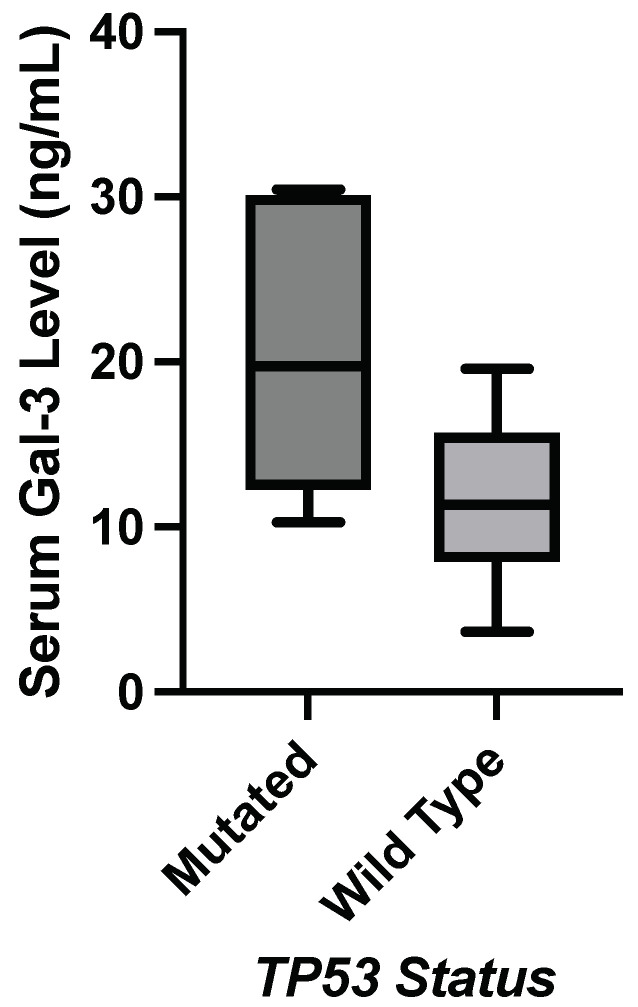
Nonparametric testing found statistically significant elevation in galectin-3 levels in Luminal A subtype patients with a *TP53* mutation (*p*-value = 0.0184).

**Figure 4:**
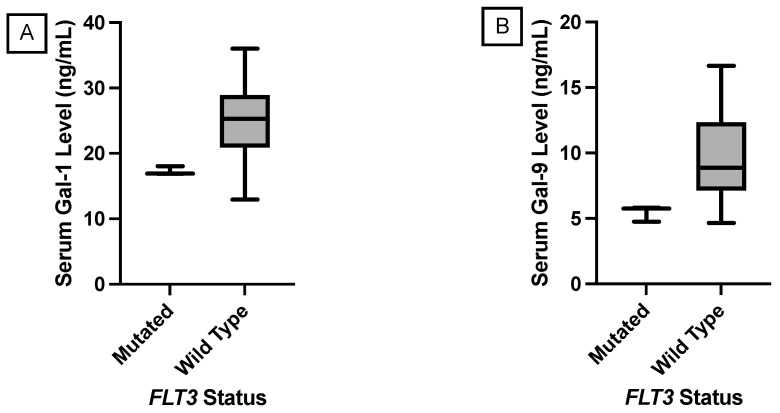
Nonparametric comparison of serum galectin levels of breast cancer patients with the Luminal A molecular subtype. (**A**) Serum galectin-1 levels were significantly lower in patients with a *FLT3* mutation (*p*-value = 0.0296). (**B**) Serum galectin-9 levels were significantly lower in patients with a *FLT3* mutation (*p*-value = 0.0362).

Table 6 shows the *p*-value comparisons of mean serum galectin levels and the presence of a hotspot mutation in *TP53*, *MET*, and *FLT3* genes. Figure 3 shows a significant correlation between a *TP53* mutation and an elevation in median serum galectin-3 levels. Figure 4 shows a significant decrease in median serum galectin-1 and -9 levels correlated with an *FLT3* mutation. This was the only significant correlation found in this study where the genetic mutation correlated with a lower galectin level. Additionally, the genetic mutation in the *FLT3* gene for these patients was found in the non-coding region compared to most of the other genetic mutations in this Hot-Spot panel, which were in the protein-coding regions of the genome. None of the Luminal A samples had a *KIT* mutation, so this correlation could not be compared with the significant findings from the larger breast cancer subset.

#### 3.4.2. Other Molecular Subtypes

The sample sizes of patients with Luminal B, Luminal A HER2 Hybrid, Luminal B HER2 Hybrid, and HER2 Positive subtypes were too small to perform a *t*-test that would provide reliable results. A Wilcoxon Rank Sum Test was also performed for samples with the Triple-Negative subtype, but no statistical difference in galectin-1, -3, and -9 levels was found.

Table 7 shows the *p*-values for Wilcoxon Rank Sum Test comparisons of serum galectin levels and the presence of a hotspot mutation in specific cancer-critical genes. None of the *p*-values were statistically significant. The number of mutated samples is out of twelve samples that were classified as Triple-Negative.

### 3.5. Histologies

Breast cancer histologies are determined based on the location of tumor origin and the presence or absence of spread to the stroma of the breast [23]. Invasive ductal carcinoma is the most common breast cancer histology and accounts for twenty-five samples in this study (35.2%). Ductal carcinoma in situ is the second most common breast cancer histology, accounting for twenty patients in this study (28.2%). Ductal carcinoma in situ is confined to the mammary ducts, while invasive ductal carcinoma signifies that the cancer has spread to the stroma as well. Patients’ ductal carcinoma in situ can become invasive over time as the disease progresses [24]. Histologies in this study represent the diagnosis at the time of sample retrieval.

No significant differences in mean serum galectin levels were found in the presence of specific cancer-critical gene mutations in either the ductal carcinoma in situ or invasive ductal carcinoma sample cohorts.

**Figure 5:**
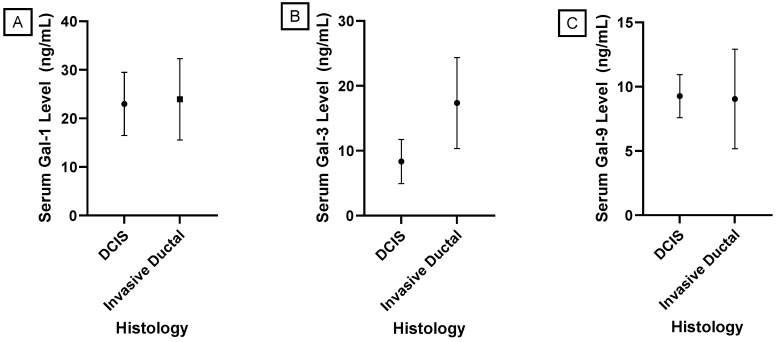
Comparison of mean serum galectin levels in patients with Ductal Carcinoma. (**A**) Mean serum galectin-1 levels were not significantly different between patients with invasive ductal carcinoma and DCIS (*p*-value = 0.6799). (**B**) Mean serum galectin-3 levels were significantly elevated in patients with invasive ductal carcinoma (*p*-value < 0.0001). (**C**) Mean serum galectin-9 levels were not significantly different between patients with invasive ductal carcinoma and DCIS (*p*-value = 0.8192).

A Levene’s Test for Homogeneity of Variance was performed, indicating a statistically significant difference in variances between the samples from patients with invasive ductal carcinoma and the samples from patients with ductal carcinoma in situ for galectin-3 and -9. Based on these findings, a pooled *t*-test for differences in means was performed to compare mean serum galectin-1 levels between invasive ductal carcinoma and ductal carcinoma in situ, while a Welch’s *t*-test was performed for galectin-3 and -9. Figure 5 shows that serum galectin-3 levels were significantly elevated in patients with invasive ductal carcinoma with a *p*-value < 0.0001. There was no significant correlation found between histology and mean serum galectin-1 or -9 levels with *p*-values of 0.6799 and 0.8192, respectively.

### 3.6. Stages

When comparing galectin levels between stages, no significant difference was found in median serum galectin-1, -3, and -9 levels between stages. Previous studies have also found a lack of correlation between specific breast cancer stages and the dysregulation of serum galectin levels, but instead there is an upregulation of serum galectin levels across breast cancer stages [25]. Additionally, our analysis found no significant differences in correlation between specific genetic mutations and median serum galectin levels within each stage when using a Wilcoxon Rank Sum Test.

### 3.7. Gene Combinations

#### 3.7.1. ANOVA

The following analyses showed that specific combinations of genetic mutations modulated galectin levels even when the constituent gene mutations alone did not result in any significant difference in mean serum galectin levels. ANOVA was performed between samples with both a *PIK3CA* and a *TP53* mutation (*n* = 7), a *PIK3CA* mutation but no *TP53* mutation (*n* = 24), a *TP53* mutation but no *PIK3CA* mutation (*n* = 16), and neither mutation (*n* = 24) found a *F*-statistic = 10.64 and a *p*-value < 0.0001. This significant *F*-statistic indicates a significant difference in at least one of the mean serum galectin-3 levels. Additionally, the adjusted $R^{2}$ = 0.292, so approximately 29.2% of the variance in serum galectin-3 levels can be attributed to this combination of mutations while adjusting for multiple testing error.

#### 3.7.2. Steel–Dwass Test

Because of the small sample sizes, a Steel–Dwass test was performed comparing medians between samples as a nonparametric test while accounting for Type I error from multiple tests. Patients with both a *PIK3CA* and a *TP53* mutation had significantly elevated median serum galectin-3 levels compared to patients with a *PIK3CA* mutation and the wild-type *TP53* gene with a *p*-value = 0.0192. Patients with both a *PIK3CA* and a *TP53* mutation had significantly elevated median serum galectin-3 levels compared to patients with a *TP53* mutation and the wild-type *PIK3CA* gene with a *p*-value = 0.0344. Patients with both a *PIK3CA* mutation and a *TP53* mutation had significantly elevated median serum galectin-3 levels compared to patients with wild-type *PIK3CA* and *TP53* genes with a *p*-value = 0.0254. This supports a significant correlation between the presence of both a *PIK3CA* and a *TP53* mutation and elevated serum galectin-3 levels.

**Figure 6:**
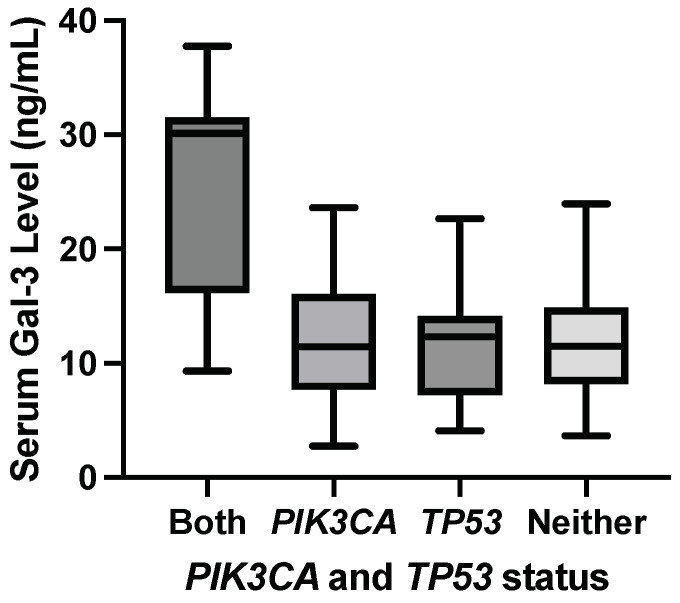
Median serum galectin-3 levels were significantly elevated in samples with both a *PIK3CA* hotspot mutation and a *TP53* hotspot mutation compared to samples with a *PIK3CA* mutation but no *TP53* mutation, a *TP53* mutation but no *PIK3CA* mutation, and samples with neither mutation.

Figure 6 shows the elevation in median serum galectin-3 level for samples with both a *PIK3CA* hotspot mutation and a *TP53* hotspot mutation compared to samples with a *PIK3CA* mutation but no *TP53* mutation, a *TP53* mutation but no *PIK3CA* mutation, and samples with neither mutation.

### 3.8. Metastasis

Table 8 shows that the odds of metastasis in a patient with a *KIT* mutation is 11.20 times the odds of metastasis in patients without a *KIT* mutation.

Table 9 shows that the odds of metastasis in a patient with a *PIK3CA* mutation is 2.43 times the odds of metastasis in patients without a *PIK3CA* mutation.

## 4. Discussion

### 4.1. Study Findings

This study found a significant correlation between a cancer-critical *KIT* mutation and higher serum galectin-9 levels in breast cancer patients, similar to other studies [21]. While the mechanisms behind galectin elevations with *KIT* mutations are still being explored, one possible reason is that increased RTK/RAS signaling through gain-of-function mutations promotes the transcription of these galectins downstream in the signaling pathway. This has been demonstrated by galectin-3 and the transcription factor FOXD1 forming a positive feedback loop through the ERK intracellular signaling pathway, which promotes lung cancer aggressiveness [26].

This study analyzed specific molecular subtypes and found that patients within the Luminal A subtype and with a *TP53* mutation had significantly elevated median serum galectin-3 levels compared to Luminal A samples without a *TP53* mutation. This result aligns with other studies that indicate that the upregulation of galectin-3 is associated with a loss in p53-induced apoptosis [27]. Specifically, one study found that the loss of the p53 activator HIPK2 resulted in galectin-3 upregulation [28]. This loss of function in the p53 signaling pathway that leads to a loss in regulation over galectin-3 expression provides a possible explanation for the elevated galectin-3 levels that were observed with *TP53* mutations.

While previous studies have found a higher proportion of *TP53* mutations within TNBC, this study did not find a significant correlation between the *TP53* genetic mutation and a difference in galectin levels [29,30]. The rationale for this finding is likely the limited sample size of patients with *TP53* mutations and TNBC. Unlike the other results found in this study where genetic mutations correlate with elevated galectin levels, an *FLT3* mutation correlated with significantly lower galectin-1 and -9 levels in patients with the Luminal A subtype. Considering the role of *FLT3* in the RTK/RAS pathway, it would be expected that a gain-of-function mutation would result in the upregulation of galectin-1 and -9 similar to the observations with *KIT* and *MET* mutations. Perhaps this downregulation is part of a broader reprogramming of the tumor microenvironment that is not fully understood, which is why we recommend the relationship between galectins and *FLT3* be further explored. No significant correlations between genetic mutations and galectin levels were found in patients with the Triple-Negative subtype. While other molecular subtypes were represented in this study, there was not a large enough sample size within these subtypes to accurately analyze them. These findings suggest that the specific molecular subtype is important when considering therapy choices as different genes are implicated in galectin modulation depending on the subtype. A combination of disease subtype and genetic screening may also foreshadow the resulting galectin levels and subsequently disease progression.

Patients with a diagnosis of invasive ductal carcinoma at the time of sample retrieval had significantly elevated mean galectin-3 levels compared to patients with ductal carcinoma in situ histology. However, no significant correlations between genetic mutations and galectin levels were found within each ductal histology.

Mutations in the *PIK3CA* gene and the *KIT* gene are heavily implicated in regulating metastasis based on the respective odds ratios of 11.20 and 2.43 found in this study. This association between a cancer-critical mutation in the *KIT* gene and elevated odds of metastatic disease is supported by previous findings [21]. However, no significant correlations between genetic mutations and galectin levels were found within the primary and metastatic breast cancer subsets.

### 4.2. Limitations

While this study revealed very promising and clinically relevant findings, it is not without limitations. The major limitation is the sample size. The sample set was determined based on the availability of the PHCI’s biorepository of cancer patients who had also received a complete hotspot panel of cancer-critical genes. While a sample size of seventy-one is over double the sample size other recent studies that compare genetic mutations and galectin levels, an analysis of a larger sample would solidify the findings of this study and potentially identify more correlations between genetic mutations and galectin levels that were labeled as borderline in this study. Additionally, a larger sample size would provide the opportunity to analyze molecular subtypes and histologies that were omitted in this study due to a lack of sufficient samples.

### 4.3. Next Steps

When more samples become available, this analysis should be repeated to improve the power of these findings. Analyzing the effect of genetic mutations in cancer-critical genes on the regulation of galectin levels sets the framework for examining the specific mechanisms by which galectin levels are altered and how this impacts cancer outcomes. These pathways are important for developing effective galectin-targeted therapies. To examine the manner in which galectins are altered during the course of disease progression, it would be helpful to measure serum galectin levels over the disease course in the same patients. Patients with and without specific genetic mutations should be compared. Furthermore, the interaction between different genetic mutations in regulating galectin levels is a new finding in this correlation analysis study, and a mechanistic analysis should be performed to understand these interactions and their implications for novel therapies.

While this study focused on analyzing galectin-1, -3, and -9, examining other galectin types is a potential route for further analysis. For instance, galectin-7 overexpression has been shown to significantly increase the ability of breast cancer cells to metastasize to lungs and bones [31]. One study suggests that mutated p53 induces the overexpression of galectin-7, and further analysis could elucidate the relationship of galectin-7 with *TP53* and other gene mutations [32]. Additionally, elevated galectin-4 expression has been associated with numerous cancer types, contributing to cancer pathogenesis through a variety of mechanisms ranging from increased metastatic behavior to the downregulation of pro-apoptotic proteins p21 and Bax [33,34]. Incorporating a broader spectrum of galectin types into future investigations could unveil crucial insight into their relationships with cancer-driving mutations.

## Figures and Tables

**Table 1:** Galectin serum concentration summary.

Galectin	*n*	Mean (ng/mL)	SD (ng/mL)	Median (ng/mL)	IQR (ng/mL)	Min–Max (ng/mL)
Gal-1	71	23.77	7.06	23.61	17.70–27.78	11.02–46.09
Gal-3	71	13.31	6.86	12.24	8.68–16.39	2.76–37.75
Gal-9	50	9.17	3.23	9.14	6.37–11.16	3.56–17.96

**Table 2:** Mutation counts with specific amino acid substitutions.

Gene	Sample Count	Mutation	Mutation Count	Percent of Mutations
*PIK3CA*	31	p.Ile391Met	10	7.87%
		p.His1047Arg	10	7.87%
		p.Glu545Lys	7	5.51%
		p.Glu542Lys	2	1.57%
		Other	5	3.94%
		Total	34	26.77%
*TP53*	23	p.Pro72Arg	5	3.94%
		p.Arg273His	2	1.57%
		Other	20	15.75%
		Total	27	21.26%
*KDR*	19	p.Gln472His	19	14.96%
		Total	19	14.96%
*MET*	8	p.Asn375Ser	6	4.72%
		p.Met362Thr	2	1.57%
		Total	8	6.30%
*KIT*	6	p.Met541Leu	5	3.94%
		p.Val530Ile	1	0.79%
		Total	6	4.72%
Other	27	Other	33	25.98%
Total	114		127	

**Table 3:** *PIK3CA*, *TP53*, and *KDR* mutation status compared to galectin levels.

			Mean Galectin		
			Concentration (SD), ng/mL		
Gene	Count	Galectin	Wild-Type	Mutated	*p*-Value
*PIK3CA*	31	Gal-1	23.67 (6.25)	23.89 (8.10)	0.8976
		Gal-3	12.07 (5.02)	14.92 (8.51)	0.1046 *
		Gal-9	8.98 (2.97)	9.43 (3.62)	0.6305
*TP53*	23	Gal-1	23.91 (6.32)	23.47 (8.56)	0.8114
		Gal-3	12.07 (5.07)	15.90 (9.19)	0.0724 *
		Gal-9	9.18 (2.94)	9.16 (3.75)	0.9855
*KDR*	19	Gal-1	24.06 (7.06)	22.96 (7.21)	0.5651
		Gal-3	12.69 (6.42)	15.03 (7.87)	0.2049
		Gal-9	8.97 (3.12)	9.60 (3.54)	0.5275

* denotes the use of a Welch’s Test in place of a pooled *t*-test as indicated by a Levene’s Test.

**Table 4:** *MET* and *KIT* mutation status compared to galectin levels.

			Median Galectin		
			Concentration (IQR), ng/mL		
Gene	Count	Galectin	Wild-Type	Mutated	*p*-Value
*MET*	8	Gal-1	23.74 (17.90–27.78)	23.51 (15.43–29.06)	0.5304
		Gal-3	11.85 (8.94–16.16)	13.16 (6.66–17.63)	0.9493
		Gal-9	9.04 (6.37–10.90)	11.22 (6.23–13.45)	0.3343
*KIT*	6	Gal-1	23.61 (17.04–27.63)	29.16 (21.84–39.12)	0.1003
		Gal-3	11.85 (8.37–16.28)	13.18 (9.40–33.07)	0.3685
		Gal-9	8.88 (6.05–10.96)	10.57 (9.95–15.27)	0.0485

Statistically significant findings (*p*-value < 0.05) are highlighted in red.

**Table 5:** Luminal A: *PIK3CA* and *KDR* mutation status compared with galectin levels.

			Mean Galectin		
			Concentration (SD), ng/mL		
Gene	Count	Galectin	Wild-Type	Mutated	*p*-Value
*PIK3CA*	20	Gal-1	23.31 (4.59)	24.87 (7.25)	0.4592
		Gal-3	11.36 (4.79)	14.73 (6.97)	0.1084
		Gal-9	8.67 (3.64)	9.41 (3.11)	0.5931
*KDR*	10	Gal-1	23.86 (5.45)	24.89 (7.85)	0.6502
		Gal-3	12.07 (5.71)	13.59 (7.65)	0.8224
		Gal-9	8.27 (2.80)	10.66 (3.86)	0.0958

**Table 6:** Luminal A: *TP53*, *MET*, and *FLT3* mutation status compared with galectin levels.

			Median Galectin		
			Concentration (IQR), ng/mL		
Gene	Count	Galectin	Wild-Type	Mutated	*p*-Value
*TP53*	7	Gal-1	24.70 (19.68–27.76)	24.97 (16.93–31.76)	1.0000
		Gal-3	11.36 (7.89–12.24)	19.75 (12.24–30.11)	0.0014
		Gal-9	8.63 (6.64–12.08)	7.70 (5.76–12.98)	0.7269
*MET*	5	Gal-1	24.97 (18.59–27.67)	24.70 (18.28–30.54)	0.8548
		Gal-3	12.67(5.93–18.25)	11.85 (8.94–16.56)	0.9271
		Gal-9	7.96 (5.80–11.97)	14.24 (11.82–16.66)	0.1055
*FLT3*	3	Gal-1	25.28 (20.93–28.93)	(16.88–18.04)	0.0296
		Gal-3	11.85 (7.99–16.73)	12.24 (10.28–19.75)	0.6470
		Gal-9	8.88 (7.14–12.34)	5.76 (4.76–5.82)	0.0362

Statistically significant findings (*p*-value < 0.05) are highlighted in red.

**Table 7:** TNBC: *TP53*, *PIK3CA*, *KDR*, and *KIT* mutation status compared with galectin levels.

			Median Galectin		
			Concentration (IQR), ng/mL		
Gene	Count	Galectin	Wild-Type	Mutated	*p*-Value
*TP53*	6	Gal-1	19.73 (14.19–24.08)	24.73 (17.95–29.72)	0.2980
		Gal-3	9.88 (6.32–14.79)	11.88 (7.53–15.30)	0.6889
		Gal-9	8.00 (4.69–10.36)	10.57 (7.94–10.96)	0.2703
*PIK3CA*	4	Gal-1	23.07 (17.04–28.54)	19.21 (12.08–25.35)	0.3502
		Gal-3	11.88 (7.80–16.62)	9.88 (4.34–12.99)	0.5522
		Gal-9	10.57 (6.40–10.84)	6.86 (4.12–9.60)	0.1877
*KDR*	4	Gal-1	23.29 (17.11–26.57)	17.81 (14.44–26.69)	0.5522
		Gal-3	9.88 (5.23–13.15)	12.64 (8.73–20.19)	0.3502
		Gal-9	10.39 (6.86–10.85)	8.48 (5.71–10.77)	0.9025
*KIT*	2	Gal-1	23.07 (14.89–27.34)	21.25 (19.33–23.17)	0.9145
		Gal-3	11.00 (8.38–14.79)	7.81 (2.76–12.85)	0.5912
		Gal-9	10.39 (5.38–10.84)	10.08 (9.60–10.57)	1.0000

**Table 8:** *KIT* mutation frequency in primary and metastatic breast cancer samples.

*KIT* Status	Metastatic Count	Primary Count
Mutated	3	3
Wild-Type	5	36

**Table 9:** *PIK3CA* mutation frequency in primary and metastatic breast cancer samples.

*PIK3CA* Status	Metastatic Count	Primary Count
Mutated	5	24
Wild-Type	3	35

## Data Availability

The data presented in this study are available in this article.

## Supplementary Materials

The following supporting information can be downloaded at https://www.mdpi.com/article/10.3390/genes15060818/s1, Table S1: Galectin Concentrations vs. Mutations Data.

## Abbreviations

The following abbreviations are used in this manuscript:

TNBC	Triple-Negative Breast Cancer
RTK	Receptor Tyrosine Kinase
ELISA	Enzyme-linked Immunosorbent Assay
PCR	Polymerase Chain Reaction
PHCI	Prisma Health Cancer Institute’s Biorepository
HER2	Human Epidermal Growth Factor Receptor 2
EGFR	Epidermal Growth Factor Receptor
ALK	Anaplastic Lymphoma Kinase
PDGFR	Platelet-Derived Growth Factor Receptor
TGF-βR	Transforming Growth Factor Beta
KIT	Mast/Stem Cell Growth Factor Receptor KIT
TP53	EKC/KEOPS Complex Subunit TP53RK
FLT3	Fibroblast Growth Factor Receptor
PIK3CA	Phosphatidylinositol 4;5-Bisphosphate 3-Kinase Catalytic Subunit Alpha Isoform
PTEN	Phosphatase and Tensin Homolog
CDH1	Cadherin 1
STK11	Serine/Threonine Kinase
BRCA1	Breast Cancer Gene 1
BRCA 2	Breast Cancer Gene 2
ATM	Ataxia Telangiectasia Mutated
CHEK2	Checkpoint kinase 2
BRIP1	BRCA1 Interacting Protein
MET	Hepatocyte Growth Factor Receptor
VEGF	Vascular Endothelial Growth Factor
DDR1	Discoidin Domain Receptor 1
EphA10	Ephrin Receptor A10
BCR-ABL	Breakpoint Cluster Region-Abelson Murine Leukemia Viral Oncogene Homolog 1
KDR	Vascular Endothelial Growth Factor Receptor 2
ANOVA	Analysis of Variance
RAS	Rat Sarcoma
FOXD1	Forkhead Box D1
ERK	Extracellular Signal-Regulated Kinases
Gal-1	Galectin-1
Gal-3	Galectin-3
Gal-9	Galectin-9
FLT3	FMS-like Tyrosine Kinase 3
